# Detection of *Bartonella* spp. in fleas by MALDI-TOF MS

**DOI:** 10.1371/journal.pntd.0006189

**Published:** 2018-02-16

**Authors:** Basma El Hamzaoui, Maureen Laroche, Lionel Almeras, Jean-Michel Bérenger, Didier Raoult, Philippe Parola

**Affiliations:** 1 Aix Marseille Univ, IRD, AP-HM, SSA, VITROME, IHU-Méditerranée Infection. 19–21 Boulevard Jean Moulin, Marseille, France; 2 Unité de Parasitologie et entomologie, Département des maladies infectieuses, Institut de Recherche Biomédicale des Armées, IHU Méditerranée Infection, Marseille, France; University of Tennessee, UNITED STATES

## Abstract

**Background:**

Matrix-Assisted Laser Desorption/Ionization Time-of-Flight Mass Spectrometry (MALDI-TOF MS) has recently emerged in the field of entomology as a promising method for the identification of arthropods and the detection of associated pathogens.

**Methodology/Principal findings:**

An experimental model of *Ctenocephalides felis* (cat fleas) infected with *Bartonella quintana* and *Bartonella henselae* was developed to evaluate the efficacy of MALDI-TOF MS in distinguishing infected from uninfected fleas, and its ability to distinguish fleas infected with *Bartonella quintana* from fleas infected with *Bartonella henselae*. For *B*. *quintana*, two groups of fleas received three successive blood meals, infected or not. A total of 140 fleas (100 exposed fleas and 40 control fleas) were engorged on human blood, infected or uninfected with *B*. *quintana*. Regarding the second pathogen, two groups of fleas (200 exposed fleas and 40 control fleas) were fed in the same manner with human blood, infected or not with *Bartonella henselae*. Fleas were dissected longitudinally; one-half was used for assessment of *B*. *quintana* and *B*. *henselae* infectious status by real-time PCR, and the second half was subjected to MALDI-TOF MS analysis. Comparison of MS spectra from infected fleas and uninfected fleas revealed distinct MS profiles. Blind queries against our MALDI-TOF MS arthropod database, upgraded with reference spectra from *B*. *quintana* and *B*. *henselae* infected fleas but also non-infected fleas, provided the correct classification for 100% of the different categories of specimens tested on the first model of flea infection with *Bartonella quintana*. As for *Bartonella henselae*, 81% of exposed qPCR-positive fleas, 96% of exposed qPCR-negative fleas and 100% of control fleas were correctly identified on the second model of flea infection.

MALDI-TOF MS successfully differentiated *Bartonella* spp.-infected and uninfected fleas and was also able to correctly differentiate fleas infected with *Bartonella quintana* and fleas infected with *Bartonella henselae*. MALDI-TOF MS correctly identified flea species as well as their infectious status, consistent with the results of real-time PCR.

**Conclusions/Significance:**

MALDI-TOF is a promising tool for identification of the infection status of fleas infected with *Bartonella* spp., which allows new possibilities for fast and accurate diagnosis in medical entomology and vector surveillance.

## Author summary

Fleas are known vectors of human infectious diseases. Identification of fleas and their associated pathogens is essential for the prevention of flea-borne diseases. Currently, the morphological identification of arthropods based on dichotomous keys, as well as molecular techniques, are the most common approaches for arthropod identification and entomological surveillance. In recent years, MALDI-TOF MS has revolutionized clinical microbiology in enabling the rapid identification of bacteria and fungi by comparing the protein profiles obtained to a database. This proteomic approach has recently been used for arthropod identification and pathogen detection. Here, we developed an experimental model to test MALDI-TOF's ability to differentiate fleas infected with human pathogens, *Bartonella quintana* and *Bartonella henselae*, from uninfected fleas.

## Introduction

Fleas are wingless insects characterized by a laterally flattened body between 1.5 and 4 mm long. Males and females are strict hematophagous parasites of mammals and birds [1].

Fleas are vectors of human infectious diseases such as bubonic plague, caused by *Yersinia pestis*, and murine typhus, caused by *Rickettsia typhi* [2,3]. Fleas can also transmit *Bartonella henselae*, the agent of cat-scratch disease. Recently, *Ctenocephalides felis*, the cat flea, was described as a potential vector of *Bartonella quintana*, the agent of trench fever, known to be transmitted by the human body louse *(Pediculus humanus humanus)*[4,5].

Currently, the most widely used method for flea identification is based on morphological criteria [6]. This approach relies on the use of identification keys that require entomological expertise and specific documentation. However, the number of entomologists specialized in flea’s taxonomy is very small [7]. Also, the accurate identification of most flea species requires specimens to be slide mounted for stereoscopic examination, which makes them unusable for further molecular studies [8].

During entomological surveys, the determination of the infection status of fleas is also a critical step in assessing the risk of disease transmission. Molecular biology has been used in the last 20 years for arthropod identification and detection of their associated pathogens [8]. However, this approach is limited by the availability of reference sequences in the GenBank database. The length of time and the cost associated with molecular biology approaches can be limiting as well.

Recently, Matrix-Assisted Laser Desorption/Ionization Time-Of-Flight Mass Spectrometry (MALDI-TOF MS), a technique based on the analysis of protein fingerprints, has revolutionized clinical microbiology, enabling the rapid identification of bacteria and fungi by comparing their protein profiles to a reference database [9]. More recently, MALDI-TOF MS has been successfully applied to the identification of arthropods, including fleas [8,10]. Moreover, this tool has been tested for its ability to detect pathogens in arthropods, and it has been reported that MALDI-TOF is able to successfully differentiate ticks infected or uninfected by *Borrelia* or *Rickettsia* spp. [11,12].

In this context, we set up an experimental model of flea infection with *B*. *quintana* and *Bartonella henselae*. We ranked the flea groups according to their infection status as confirmed with molecular biology, and we evaluated MALDI-TOF's ability to distinguish the different groups and different pathogens.

## Materials and methods

### Flea rearing

Cat fleas (*Ctenocephalides felis)* were maintained under standard laboratory rearing conditions as previously described [4]. Adult stages were fed with human blood previously heated in a water bath at 37°C for 30 minutes. It was made available to the fleas via glass tubes closed with parafilm (Sigma-Aldrich, Saint Louis, Missouri, USA), which were fixed to the plates containing the fleas [4]. The breeding cells containing the fleas are in contact with the lower part of the membrane. The eggs are collected with adult feces 1–2 times a week in sterile tubes containing 10 grams of larval nutrient medium, composed of 20 g rodent food, 40 g sand, 3 g lyophilized blood, and 2 g yeast. The tubes were placed in the laboratory incubator in the same temperature and humidity conditions used for the adults (80% humidity, 27°C). The device is kept in the dark until the emergence of the adult stages [4].

### *Bartonella quintana* and *Bartonella henselae*

All experiments conducted with *Bartonella quintana* and *Bartonella henselae* were performed inside a Biosafety cabinet in a Biosafety Level 2 (BSL2) room. The two strains of bacteria (*B*. *quintana* strain Oklahoma ATCC 49793, *Bartonella henselae* strain Marseille) were grown on Columbia sheep blood agar plates (5%, BioMerieux, Marcy l’Etoile, France) at 37°C in atmosphere enriched with 5% CO_2_ [13]. After 10 days of culturing, the bacteria were collected and transferred to a tube containing 400 μL of phosphate buffered saline (PBS), pH 7.2 (PBS, BioMerieux, Marcy l’Etoile, France). Two hundred microliters of this bacterial suspension mixed with 2 mL of human blood, like a simple mixture of *Bartonella* sp. and erythrocytes not an erythrocyte infection, were used for artificial infection of fleas. An aliquot of 2 ml of human whole blood containing 200 μl of PBS was used to feed the control group. The bacterial suspension (100 μl) was used for bacterial quantification by flow cytometry. The inocula were marked by DAPI (4',6-diamidino-2-phénylindole) and counting beads to quantify the number of bacteria. The absolute number of cells of interest (cells/μL) was calculated according to the manufacturer’s recommendations. The last 100 μl of inoculum was used for bacterial culture [14].

### Artificial infection of fleas

For secure experimentation, a Georgie and Wade confined equipment (BSL3) was used for flea infection with *B*. *quintana* and *B*. *henselae* [4]. It is composed of a glove box with a safety airlock to load and unload material. For artificial infection with *Bartonella quintana*, two groups were created, an exposed group containing 100 *Ctenocephalides felis* fleas (40 males and 60 females) and a control group containing 40 *Ctenocephalides felis* fleas (20 males and 20 females). For artificial infection with *Bartonella henselae*, two groups were formed; an exposed group containing 200 *Ctenocephalides felis* fleas (120 females and 80 males) and a control group containing 40 *Ctenocephalides felis* fleas (20 males and 20 females). Each flea group received three successive human blood meals. Control fleas were fed with *Bartonella-*free blood mixed with 200 μL of PBS and exposed fleas were fed with blood infected with 200 μL of bacterial suspension [15]. These meals were delivered every 48 hours [4].

### DNA extraction and PCR detection of *Bartonella quintana* and *Bartonella henselae*

Three days after the last infective meal, ten fleas from each group were subsequently collected every two days. They were washed individually with 70% ethanol, and then rinsed twice with distilled water. Each flea was dissected using a sterile scalpel. Half of the body was selected for real-time PCR detection of the presence of *Bartonella quintana* and *Bartonella henselae* and the rest of the body was saved for MALDI-TOF MS analysis. Flea samples destined for molecular biology were incubated overnight at 56°C in 180 μl of G2 buffer (Qiagen, Hilden, Germany) (30 mM Tris-Cl, 30 mMEDTA; 5% Tween 20, 0.5% Triton X-100; 800 mMGuHCl) and 20 μl of proteinase K (Qiagen, Hilden, Germany) (activity of 600 mAU/ml solution or 40 mAU/mg of protein).

After lysis of the samples, DNA extraction was performed in the automatic extractor EZ1 (Qiagen, Hilden, Germany). The presence of *B*. *quintana* and *B*. *henselae* DNA in these fleas was assessed by *Bartonella-*specific real-time PCR targeting the *yopP3* gene [16] and *pap31* gene, respectively [17].

### Assessment by MALDI-TOF MS for the detection of *Bartonella quintana* and *Bartonella henselae* in fleas

The other half of the body of each flea was used to detect the presence of *Bartonella quintana* and *Bartonella henselae* in fleas by MALDI-TOF MS. The samples were ground using a TissueLyser II device (Qiagen, Hilden, Germany) in 30 μl of 50% (v/v) acetonitrile, 30 μl of 70% (v/v) formic acid (Fluka, Buchs, Switzerland) and a small amount of glass powder (Sigma, Lyon, France). They were crushed at a frequency of 30 Hz for 1 minute and this cycle was repeated 3 times, in accordance with the previously published sample preparation optimization tests [18]. After centrifugation, 1 μl of supernatant was deposited in quadruplicate on the MALDI-TOF plate and covered by 1 μl of CHCA matrix solution composed of saturated α-cyano-4-hydroxycynnamic acid (CHCA) (Sigma, Lyon, France), 50% (v/v) acetonitrile (Fluka, Buchs, Switzerland), 2.5% trifluoroacetic acid (v/v) (Aldrich, Dorset, UK) and HPLC-grade water as previously described [8].

Protein mass profiles were obtained using a Microflex LT MALDI-TOF (Bruker Daltonics, Germany) mass spectrometer, with detection in positive ion linear mode at a laser frequency of 50 Hz in a mass range 2–20 kDa. The acceleration voltage was set to 20 kV, and the time of extraction delay was 200 ns. Each spectrum corresponds to the ions obtained from 240 laser shots fired in six regions of the same deposit on the ground plate and acquired automatically using the function of the Flex Control AutoXecute V.2.4 software (Bruker Daltonics). The spectra are displayed with Flex analysis v.3.3 software and exported to ClinProTools2.2 and MALDI Biotyper v.3.0 (Bruker Daltonics, Germany) for data processing. The reproducibility of the spectra of each flea group was evaluated using the ClinProTools 2.2 software. Reference spectra (MSP, Main Spectrum Profile) were generated by the automated function of the MALDI-Biotyper software v3.0 (Bruker Daltonics, Germany) by combining the results of the spectra of at least three specimens per condition in order to create a database. MSP were produced based on an unbiased algorithm based on the peak position, intensity and frequency. Flea spectra were selected based on their reproducibility and intensity for all MS analyses, including database creation. The database was created by selecting the spectra of at least 3 to 14 specimens from infected, exposed and control groups for both trials.

### Blind tests

The ability of MALDI-TOF MS to distinguish infected, exposed but uninfected, and control fleas was evaluated by blind test analysis. After sorting the spectra, high quality and reproducible MALDI-TOF MS spectra from the half body of 8 control fleas from the first trial (*B*. *quintana)* and 2 from the second trial (*B*. *henselae*), 8 fleas infected with *B*. *quintana* and 7 fleas infected with *B*. *henselae*, and 14 fleas that fed on infected blood with *B*. *quintana* and 6 fleas that fed on infected blood with *Bartonella henselae* but tested negative in qPCR, were introduced into our in-house arthropod database (Table 1). This database already contains spectra obtained from protein extracts of the cephalothorax and legs of specimens of 4 species of fleas, including *Ctenocephalides felis felis*, *Archaeopsylla erinacei*, *Xenopsylla cheopis* and *Ctenocephalides canis*, a total of 6 tick species (*Am*. *variegatum* infected by *Rickettsia africae*, *Rh*. *sanguineus*, *Rh*. *bursa*, *Rh*. *annulatus*, *Rh*. *turanicus*, *Argas persicus*, *Hy*. *rufipes*, *Hy*. *detritum*, *I*. *ricinus*, *D*. *marginatus* and *D*. *reticulatus*), 30 mosquito species (*Anopheles gambiae* Giles, *An*. *coluzzii*, *An*. *funestus*, *An*. *ziemanni*, *An*. *arabiensis*, *An*. *wellcomei*, *An*. *rufipes*, *An*. *pharoensis*, *An*. *coustani*, *An*. *claviger*, *An*. *hyrcanus*, *An*. *maculipennis*, *Culex quinquefasciatus*, *Cx*. *pipiens*, *Cx*. *modestus*, *Cx*. *insignis*, *Cx*. *neavei*, *Aedes albopictus*, *Ae*. *excrucians*, *Ae*. *vexans*, *Ae*. *rusticus*, *Ae*. *dufouri*, *Ae*. *cinereus*, *Ae*. *fowleri*, *Ae*. *aegypti*, *Ae*. *caspius*, *Mansonia uniformis*, *Orthopodomyia reunionensis*, *Coquillettidia richiardii* and *Lutzia tigripes*,), and other arthropods, including lice (*Pediculus humanus corporis*), triatomines (*Triatoma infestans*, *Rhodnius prolixus*, *R*. *pictipes*, *R*. *robustus*, *Panstrongylus geniculatus*, *Eratyrus mucronatus*) and bedbugs (*Cimex lectularius*)[8,10,12,19–22].

**Table 1:** Results of blind test analysis after upgrading of the MALDI-TOF MS database. DB: database.

	Controls	Exposed, PCR positive (*Bartonella quintana*)	Exposed, PCR negative (*Bartonella* *quintana)*	Exposed, PCR positive (*Bartonella henselae*)	Exposed, PCR negative (*Bartonella henselae*)
Total number of spectra compared against (Blind Test)	34	33	44	30	22
Number of spectra introduced in DB	10	8	14	7	6
Percentage of accurate identification	100%	100%	100%	80%	95%

Then, after the elimination of poor quality spectra, 33 spectra of infected fleas with *Bartonella quintana*, 30 spectra of infected fleas with *Bartonella henselae*, 44 spectra of fleas that fed on infected blood but that tested negative to *Bartonella quintana* in qPCR, 22 spectra of fleas that fed on infected blood but that tested negative to *Bartonella henselae* in qPCR, and a total of 34 spectra of control fleas from the first and second trial were queried against the upgraded database (Table 1).

The results of the database queries are presented as Log Score Values (LSVs) for each spectrum, corresponding to a matched degree of signal intensities of mass spectra of the query and the reference spectra. LSVs range from 0 to 3. LSVs allow good evaluation of the reproducibility between a queried spectrum and a reference spectrum, as they are the result of a thorough comparison of peak positions and intensity between those two spectra. An LSV was obtained for each spectrum of the samples tested blindly. For each specimen, the spectrum with the highest LSVs was selected for identification [22].

### MALDI-TOF MS biomarker mass set

To appraise reproducibility of the MS profiles, spectra from the three categories (infected, exposed but PCR-negative, and control) were imported in the ClinProTools 2.2 software for both pathogens (*B*. *quintana*, *B*. *henselae*). For the determination of discriminating peak masses associated with infection status, we compared the average spectrum of the three categories of fleas. The software was used to generate a peak list for each group in the 2 to 20 kDa mass range and to identify discriminating peaks. Regarding the discrimination between control profiles and *B*. *quintana*-infected flea derived profiles, the parameter settings in ClinProTools 2.2 software for spectra preparation were as follows: a resolution of 800; a noise threshold of 2.50; a maximal peak shift of 800 ppm and a match to calibrant peaks of 20%. For the peak calculation, peak peaking was performed on single spectra with a signal-to-noise threshold of 2.50 and an aggregation of 800 ppm. As for *B*. *henselae*, the parameters were as follows: a resolution of 800; a noise threshold of 2.20; a maximal peak shift of 800 ppm and a match to calibrant peaks of 20%. For the peak calculation, peak peaking was performed on single spectra with a signal-to-noise threshold of 2.20 and an aggregation of 800 ppm. The spectra were then analyzed with the genetic algorithm (GA) model, which provided a list of discriminating peaks. Manual inspection and validation of the peaks by the operator gave a “recognition capability” (RC) value together with the highest “cross-validation” (CV) value.

## Results

### Acquisition of *Bartonella quintana* and *Bartonella henselae* by cat fleas

For *Bartonella quintana* inocula, bacterial cell numbers in the first inoculum were 8.10^6^, 7.10^6^ in the second inoculum and 7.10^6^ in the last inoculum (cells/μL), as determined by flow cytometry.

For *Bartonella henselae* inocula, bacterial cell numbers in the first inoculum were 6.10^6^, 4.10^7^ in the second inoculum, and 8.10^6^ in the last inoculum (cells/μL).

Three days after the infective blood meal, 28 control fleas and 80 *Bartonella quintana*-exposed fleas were collected for the first trial, and 20 control and 160 *Bartonella henselae*-exposed fleas were collected for the second trial. The qPCR results indicated the presence of *Bartonella quintana* DNA in 33 (41.25%) of the 80 fleas, with cycle threshold (Ct) values ranging from 27.19 to 35.85, and the presence of *Bartonella henselae* in 40 *Ctenocephalides felis* (25%) of the 160 fleas, with cycle threshold (Ct) values ranging from 28.95 to 35.80. All control fleas tested negative.

### MALDI-TOF MS detection of *Bartonella spp*. in fleas

**Fig 1:**
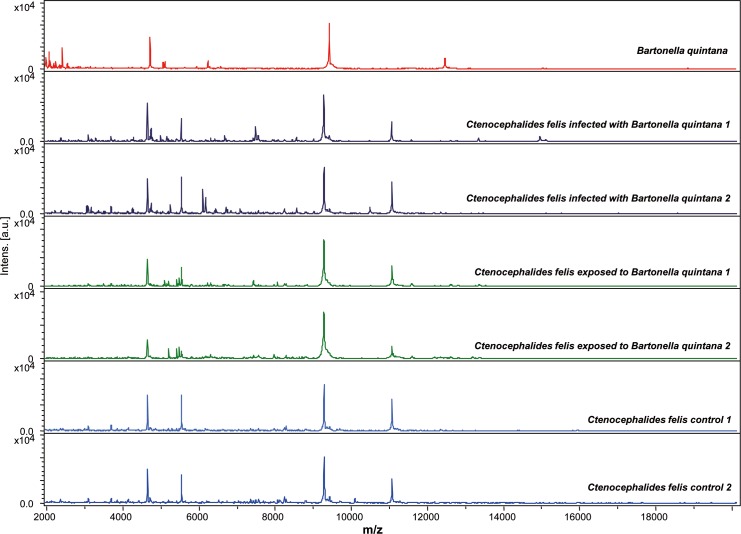
Reproducible and specific MALDI-TOF MS spectra of *Bartonella quintana* strain, body half of fresh *Ctenocephalides felis* infected with *Bartonella quintana*, exposed and control analyzed by Flex analysis 3.3 software. a.u., arbitrary units; m/z, mass-to-charge ratio.

**Fig 2:**
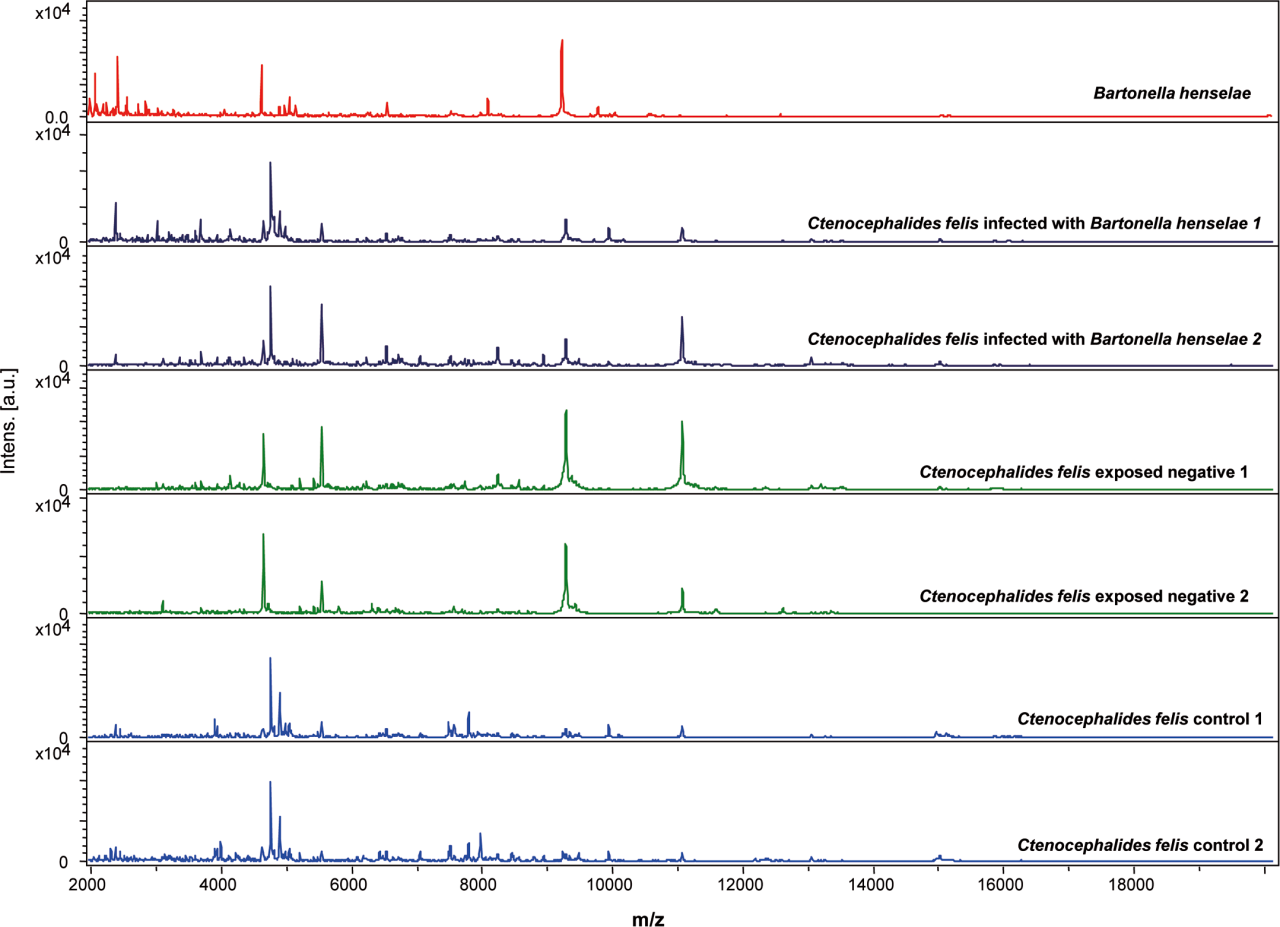
Reproducible and specific MALDI-TOF MS spectra of *Bartonella quintana* strain, body half of fresh *Ctenocephalides felis* infected with *Bartonella henselae*, exposed and control analyzed by Flex analysis 3.3 software. a.u., arbitrary units; m/z, mass-to-charge ratio.

All collected fleas were analyzed by MALDI-TOF MS. Spectra analysis with Flex analysis v.3.3 and ClinProTools 2.2 software revealed reproducibility of the profiles within the same category (Figs 1 and 2).

**Fig 3:**
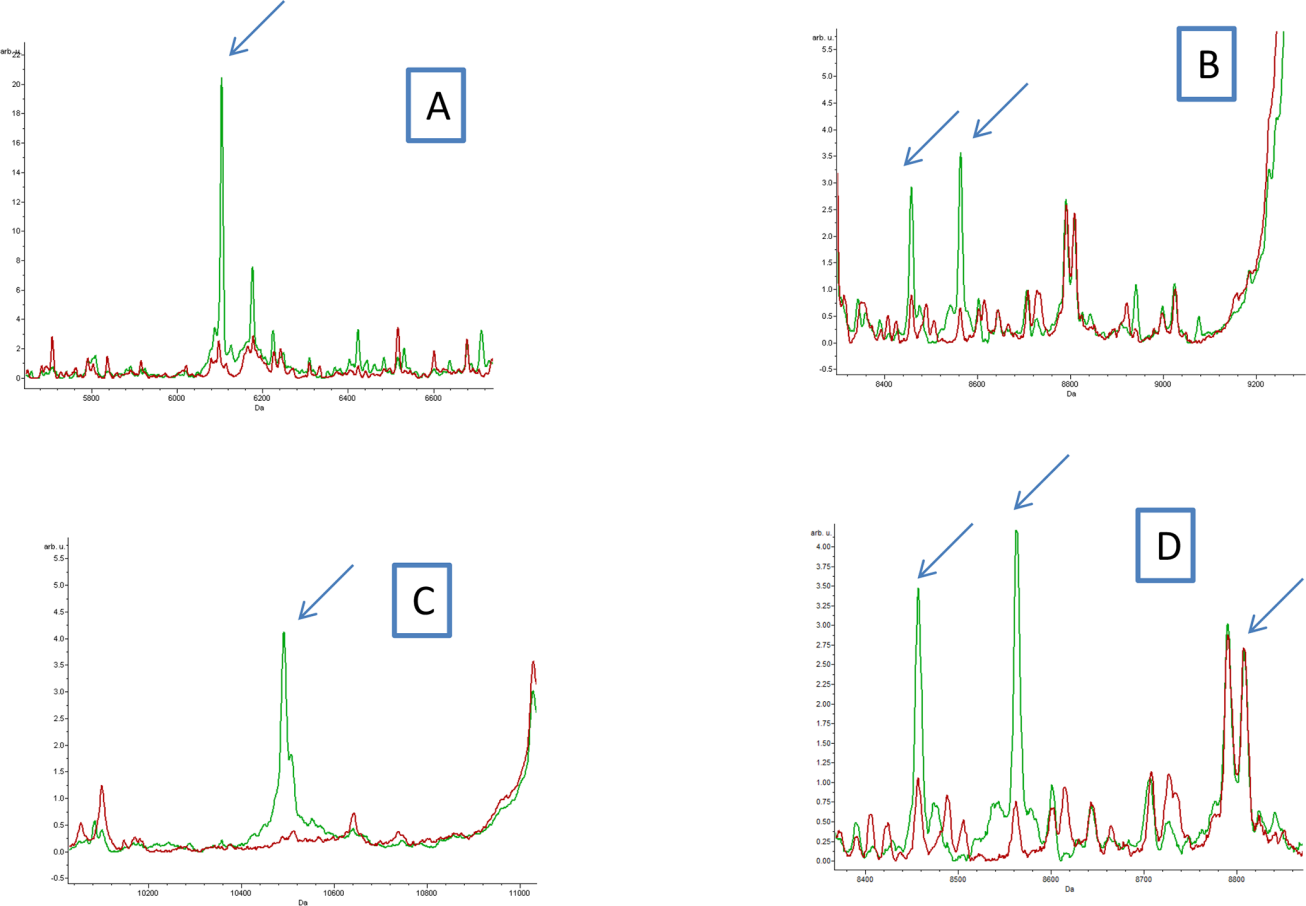
**(A, B, C and D)** Comparison of MALDI-TOF MS profiles of body half of *Ctenocephalides felis infected* or not by *Bartonella quintana* using ClinProTools 2.2 software. Red and green peaks indicated by arrows correspond to discriminating peaks of control and infected fleas respectively.

**Fig 4:**
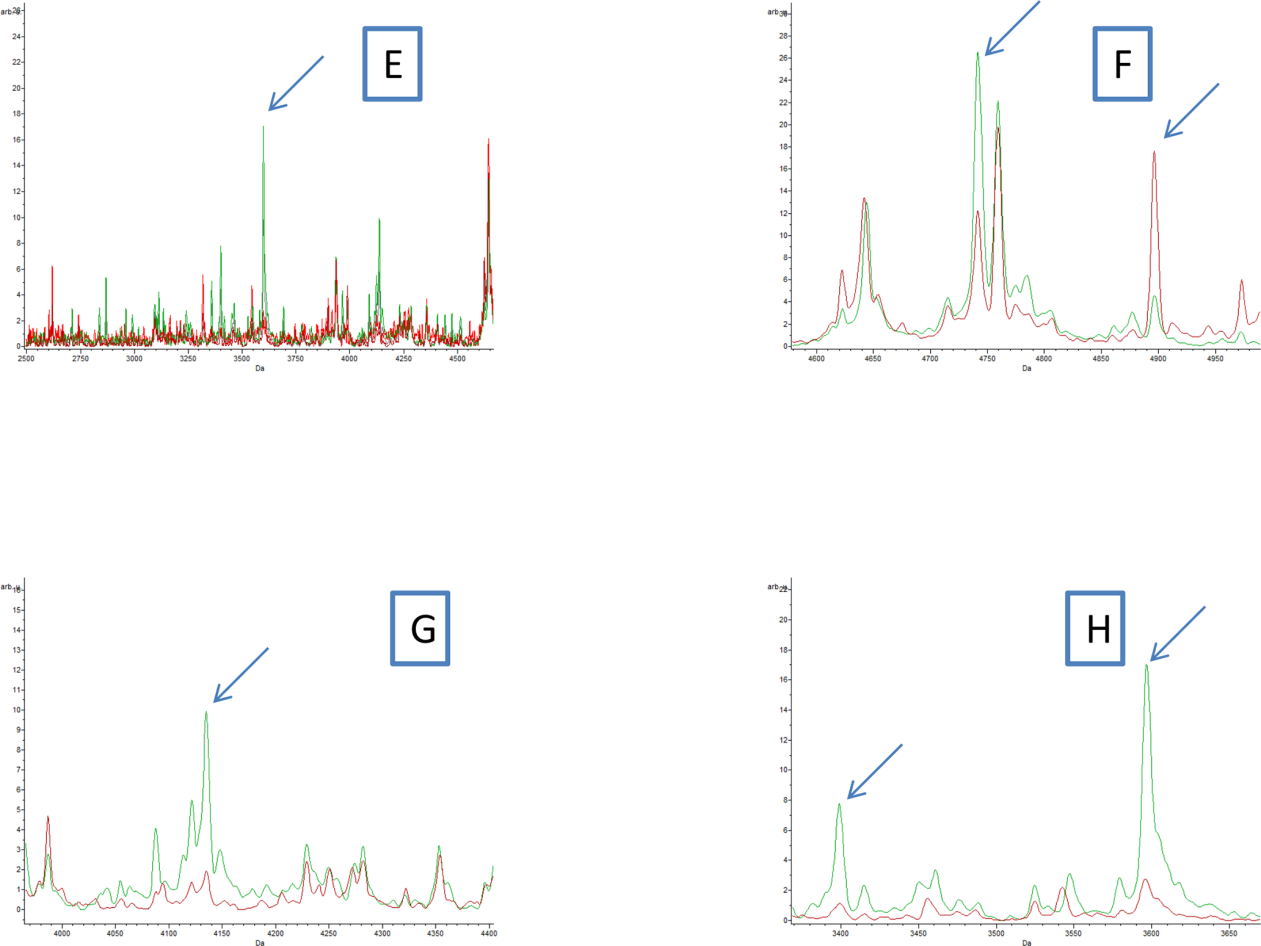
**(E, F, G and H)** Comparison of MALDI-TOF MS profiles of body half of *Ctenocephalides felis infected* or not by *Bartonella henselae* using ClinProTools 2.2 software. Red and green peaks indicated by arrows correspond to discriminating peaks of control and infected fleas, respectively.

All profiles were compared using the ClinProTools 2.2 software to appraise global reproducibility and create an average profile. Visual inspection revealed that some peaks were present in fleas infected with *Bartonella quintana* and *Bartonella henselae* profiles, but absent in control and exposed fleas (Figs 3 and 4).

The Genetic Algorithm tool of ClinProTools software was used to better identify the discriminating peaks between infected and control fleas. Spectra from 33 *Ctenocephalides felis* specimens infected with *Bartonella quintana* were compared to 22 control *Ctenocephalides felis*, same for the second pathogen; spectra from 24 *Ctenocephalides felis* specimens infected with *Bartonella henselae* were compared to 12 control *Ctenocephalides felis*. The genetic algorithm model displayed 13 peak masses that discriminated control and infected fleas with *Bartonella quintana* (Table 2), and 20 peak masses that discriminated control and infected fleas with *Bartonella henselae* (Table 3), with recognition capability (RC) and cross validation (CV) values of 100% for both comparisons.

**Table 2:** Peak masses distinguishing uninfected and *infected* fleas by *Bartonella quintana* analyzed with ClinProTools.

Mass (m/z) (Da)	*Bartonella quintana* infection status of fleas
**3963**	*Ctenocephalides felis* infected with *Bartonella quintana*
**4740**	*Ctenocephalides felis* infected with *Bartonella quintana*
**4818**	*Ctenocephalides felis* infected with *Bartonella quintana*
**5288**	*Ctenocephalides felis* control
**6422**	*Ctenocephalides felis* infected with *Bartonella quintana*
**6517**	*Ctenocephalides felis* control
**6530**	*Ctenocephalides felis* infected with *Bartonella quintana*
**6747**	*Ctenocephalides felis* control
**7039**	*Ctenocephalides felis* control
**7325**	*Ctenocephalides felis* control
**7355**	*Ctenocephalides felis* control
**8458**	*Ctenocephalides felis* infected with *Bartonella quintana*
**8563**	*Ctenocephalides felis* infected with *Bartonella quintana*

**Table 3:** Peak masses distinguishing uninfected and *infected* fleas by *Bartonella henselae* analyzed with ClinProTools.

Mass (m/z) (Da)	*Bartonella henselae* infection status of fleas
**7487.86**	*Ctenocephalides felis* infected with *Bartonella henselae*
**5478.5**	*Ctenocephalides felis* infected with *Bartonella henselae*
**5044.17**	*Ctenocephalides felis* infected with *Bartonella henselae*
**5419.73**	*Ctenocephalides felis* control
**4132.82**	*Ctenocephalides felis* infected with *Bartonella henselae*
**15023.55**	*Ctenocephalides felis* infected with *Bartonella henselae*
**9941.14**	*Ctenocephalides felis* infected with *Bartonella henselae*
**5206.22**	*Ctenocephalides felis* control
**4352.34**	*Ctenocephalides felis* control
**7737.4**	*Ctenocephalides felis* infected with *Bartonella henselae*
**7559.54**	*Ctenocephalides felis* infected with *Bartonella henselae*
**9476.91**	*Ctenocephalides felis* infected with *Bartonella henselae*
**4509.22**	*Ctenocephalides felis* infected with *Bartonella henselae*
**2616.18**	*Ctenocephalides felis* infected with *Bartonella henselae*
**6763.08**	*Ctenocephalides felis* infected with *Bartonella henselae*
**7969.85**	*Ctenocephalides felis* infected with *Bartonella henselae*
**7873.37**	*Ctenocephalides felis* infected with *Bartonella henselae*
**4897.31**	*Ctenocephalides felis* infected with *Bartonella henselae*
**13056.87**	*Ctenocephalides felis* control
**5923.83**	*Ctenocephalides felis* control

*Bartonella quintana* qPCR positive fleas (n = 33), *Bartonella henselae* qPCR positive fleas (n = 30), fleas exposed to blood infected with *B*. *quintana* but PCR negative (n = 44), fleas exposed to blood infected with *B*. *henselae* but PCR negative (n = 22) and control fleas (n = 34) were queried blindly against the MALDI-TOF arthropod database upgraded with reference spectra from these 4 category. All fleas from the artificial infection with *Bartonella quintana* were correctly identified to the species level and MALDI-TOF MS differentiated correctly the three categories of fleas (Table 1). Regarding artificial infection with *Bartonella henselae*, the identification was correct for 24/30 infected fleas (80%), 21/22 exposed negative fleas (95%) and 100% correct identification for all control fleas. For all tested samples, LSVs ranged from 2.149 to 2.859 for exposed positive fleas, from 2.091 to 2.838 for exposed negative fleas and from 2.147 to 2.713 for control fleas.

## Discussion

Recently, MALDI-TOF MS technology has been successfully used for the identification of arthropods such as fleas [10], mosquitoes [20,23], *Culicoides* [21], ticks [19,24], sand flies [25] tsetse flies [26] and triatomines [22]. The success of this method requires the standardization of sample preparation protocols to allow sharing and comparison of MS reference spectra and results between research laboratories. As is true for each innovative method, application of this tool is best with some limitations, such as the cost of the device and the comprehensiveness of the databases [23]. Furthermore, some parameters may play a role in the efficiency of MALDI-TOF MS identification, such as the conservation of arthropods after collection (18). Several recent studies have shown the efficacy of MALDI-TOF in identifying arthropods conserved in alcohol. Ticks collected in the field in Ethiopia, which were preserved in 70% ethanol for about two years, were correctly identified by MALDI-TOF. More recently, different species of ticks collected on mammals in Mali, also preserved in alcohol, were successfully identified as well by MALDI TOF, after the development of a de-alcoholization protocol providing even better results for MS identification for samples preserved in alcohol [27]. Arthropod body part selection for MALDI-TOF arthropod identification is based on the comparison of the spectra quality from different parts of the body. Flea reference spectra in our database were obtained from cephalothorax and leg protein extracts [10]. In this study, half of the body was chosen for MALDI-TOF MS and the other half for molecular biology, based on previous data indicating that the *Bartonella* species is localized in the infected flea gut tract [4]. Intraspecies reproducibility and interspecies specificity are then evaluated and the most reproducible spectra are selected to upgrade the database [8].

After the emergence of this method to identify arthropods, our research has been oriented toward the detection of associated pathogens. Preliminary encouraging work has shown that MALDI-TOF can differentiate *Rhipicephalus sanguineus* ticks infected or not by *Rickettsia conorii*, the agent of Mediterranean spotted fever [12]. It can detect *Plasmodium* parasites in anopheles [23] and *Borrelia* spp. in *Ornithodoros sonrai* ticks [11] based on spectra obtained from the cephalothorax or legs, respectively.

Here, we obtained reproducible and intense spectra from control fleas, and also from fleas infected with *B. quintana* and *B. henselae* whose infection status was previously confirmed by quantitative PCR. In these models (*B. quintana*, *B. henselae*), the bacterial loads of the infectious blood meals is higher than the bacteremia of humans with trench fever, which is around 104 to 105 [28]. However, our work focuses on the capacity of MALDI-TOF MS to distinguish fleas infected or not by *Bartonella* species. So, for this purpose we needed to obtain infected fleas. In previous published work, we have shown that in experimental models, it is difficult to infect fleas using bacteremia lower than 105 [4]. The aim of this work was not to study or explain how fleas are infected in natural cycles. This is why we chose to use higher concentrations, knowing that fleas will be successfully infected, as others have done in their models of infection [29,30]. Although the performance of the tool was assessed for the detection of the presence of *Bartonella* species in fleas, sensitivity and specificity are important parameters to consider when a new method is proposed. However, it is difficult to definitively determine the sensitivity of our method in the absence of a gold standard to determine the infectious status of fleas. While the bacterial concentration of the inoculum was measured, the exact bacterial load in each flea at the moment of the MALDI-TOF assay was unknown here. This work however was a preliminary work to determine the usefulness of MALDI TOF in differentiating infected and non-infected fleas in comparison with qPCR. For that purpose, groups were chosen regarding infection status. These groups corresponded respectively to fleas with low Ct values (below 35) for the infected group, fleas that were exposed to infected blood but had a negative qPCR test, and control fleas. Interestingly, exposed but PCR-negative fleas were classified by MALDI-TOF MS as a single category, different than infected and control categories. While all fleas were perfectly identified for each category for the *B. quintana* model, there were nevertheless limitations in the distinction between fleas infected with *B. henselae* and fleas exposed to the same bacteria but negative in qPCR, since six infected specimens were identified as exposed PCR-negative specimens. The qPCR Ct values of these specimens varied between 35.53 and 35.85; they were all collected on the first day of collection, which corresponded to the third day after the last infective blood meal. We can hypothesize that the identification of infected fleas is based on discriminating peaks associated with the flea immune response. Because this approach is strictly based on the comparison of a profile to a reference spectrum, which is a representation of the extracted global proteome of the selected body part of the flea, it is not here possible to correlate a peak position to a protein identification. To obtain such information, complementary proteomic analyses would be necessary and would help to decipher interactions between fleas and flea-borne pathogens. These peaks could be partially present in exposed fleas, perhaps temporarily, causing misidentification of these specimens as infected fleas.

A specific arthropod immune response to bacterial infection has already been described. Indeed, the production of three proteins in *Anopheles gambiae* hemolymph were increased following bacterial injection of *Escherichia coli* (XL1 Blue) and *Micrococcus luteus* (UW-Madison strain). These proteins are associated with the immune response of these mosquitoes [31]. We can therefore hypothesize that immune response proteins played a role in allowing MALDI-TOF MS to clearly distinguish between negative exposed and control fleas. During this response, some genes coding for the proteins involved in innate immunity are regulated or reduced, which could explain the disappearance of some peaks on the average profile of the control fleas [23].

The microbiome of arthropods is usually different in the field than in lab colonies [32]. It may be questioned if the microbiome of wild fleas might be more extensive and varied and might interfere with the interpretation of the MALDI TOF-MS assay. We can’t exclude the part played by the microbiome in the resulting MALDI TOF-MS spectra. Therefore, it would be interesting to study its impact on the differentiation between Bartonella-infected and uninfected fleas in future studies.

The one-step detection of the species identity of fleas and their infection status would be revolutionary for medical entomology studies, vector surveillance and movement of pathogens.
